# Medicine in China

**Published:** 1898-01

**Authors:** V. P. Suvoong

**Affiliations:** Shanghai


### MEDICINE IN CHINA.

BY V. P. SUVOONG, B. D., M. D., SHANGHAI.

(Abstract from the China Medical Missionary Journal.)

Medicine as practiced by the Chinese is in a deplorable condition. As in every department in life in China, so here also too much blind reverence is paid to old ideas. As a general thing a very few Chinese medicoes are ever educated or know anything of such a book as Ts'o-pun, an old Chinese Materia Medica, while the vast majority of them know how to write out a few old stock prescriptions copied out of some antiquated document that came down to the family as an heirloom. I know one man who was engaged in the Kiangnan Arsenal as a petty overseer, who on being dismissed from his post, was a long time out of a job; at last he made the acquaintance of a certain Buddhist priest who had written a copy of some secret prescriptions. He stole this copy, and after a few nights' application and research in that dirty dog-eared volume, he swung out a signboard informing the public that he was ready to guarantee a perfect cure for any ailment that humanity is heir to. This happened in Shanghai, and there are hundreds of similar cases where an ignoramus imposes on the public without being exposed to ridicule.

The better class of Chinese doctors have served their time in the office of their preceptors, who, however, never give lessons or readings in anything—indeed, there are no text-books to go upon. They are more like assistants, who make themselves generally useful in an office where the anxious ones look over the prescriptions and listen to whatever their chief may have to say on any given case. A Chinese medical student in the proper sense of the term has never existed. They are all charlatans, jealously guarding a few empirical ideas from the public.

One sometimes hears of the Imperial College of Medicine in Peking. A foreigner, on first hearing of it, is sure to make an egregious mistake from its high-sounding name, which leads him to suppose that it is some institution like the Royal College of Physicians and Surgeons in the West. It is only a handful of self-styled doctors like any of their brethren in Shanghai, except their good fortune has placed them in a comfortable position where they have nothing to do but to put on an air of being learned and skillful. Their anxiety commences, however, with the Imperial cough or colic. They have no lectures to give, as there are no students, and they need not write articles for medical journals, as there are no readers.

The Chinese apothecary does a large business, as can be seen from the many hongs and shops dealing in drugs in any city in China. The doctors prescribe large bowlfuls of decoctions for any complaint, so that a constantly ailing and dosing man is nick-named a "decoction pot." That is their favorite method of dispensing, though pills, powders, etc., are not neglected. Consequently a druggist must have a large supply of herbs and roots on hand to meet the demand, and he ransacks creation for the odds and ends in his shop. Of the drugs one may have some idea by looking over the advertisments in any of the Chinese newspapers published in Shanghai. I will mention one as an illustration: The advertiser in the Chen-fuh-lan-tang shop, which, has offices in Canton and elsewhere in China. A free translation of the advertisement here will show the depth of darkness in which the Chinese mind is yet enshrouded in respect of medicine:

Recently Honkong and the provinces of Kwangtung being visited by the plague, the Provinical High Authorities have published a prescription called Plague Cure which is infallible. Our shop has already prepared this medicine for the two great benevolent institutions in Canton, where it has been used with invariable success. In this prescription there is one ingredient called Son of Stone Dragon, which is found in the mountains in the province of Chekiang. Through the agency of our branch office in Hangchow we have obtained a superior variety of this stony son of a Dragon, and together with other valuable drugs we have made this mixture. During the compounding, we have reverently said a thousand prayers. Now we offer this medicine to the public. Herewith is an illustration of this stony son of a dragon as our trade mark. The medicine is not only unusually effective against the plague, but it is also infallible against different kinds of cholera, vomiting, diarrhœa, colic, apoplexy, sunstroke, asphyxia, typhus and typhoid fevers, ague, diphtheria, liver and stomach aches, tetanus in children, surfeiting, smallpox, poison, malaria, all sorts of tumors and inflammatory poisons, etc., etc. Shanghai being particular in its sanitation against plague, we have specially prepared this medicine as a valuable weapon in the hands of committees for preventive measures.

(Signed) CHEN-FUH-LAN-TANG.

I wonder who discovered such potent virtues in the little rascal of a lizard, which in its native province of Chekiang, as here, is always regarded with disfavor.

For various female complaints and diseases what are called Dragon and Phœnix Pills are largely sold in the Canton shops. These are coated with wax, either white or yellow, and of the size of an English walnut. Their efficacy is, of course, lauded to the sky, but nearly every woman that I have seen in practice has told me that she had tried them and found no improvement therefrom. No doubt they are composed of some such simple drugs as aloes, myrrh, etc., which if appropriately applied, of course have their uses; but to make them a panacea for all the complaints of women, they fail egregiously. The Chinese druggists in their anxiety to make money advertise drugs that can even restore the virile power in a profligate, and cause the sterile to bear offspring.

But there are stuffs which the Chinese druggists do conscientiously collect, and with much expense and labor, but which are nevertheless inert and useless—as tigers' bones, bears' legs, harts' horns, etc. Tigers' bones are ground into powder and used in plasters for internal injuries, bears' paws are boiled to a jelly and used as a powerful alterative for the weak and aged. Harts' horns are sawed into thin discs and boiled down and given for renewing wasted vitality. A young horn is considered as particularly valuable, when it is a little ruddy and somewhat translucent. I can not make my old friends believe that its virtue consists in ammonia, any quantity of which can be obtained almost for a song in any foreign drug-store. They rather think that I am a greenhorn myself and not particularly valuable either.

When every available drug in their reach has been pressed into service and found wanting, then they resort to superstition, or if I may be pardoned, to a "faith cure!"

Unfortunately, superstition often retains its victims long in its thraldom. But sometimes, however, it gives a sort of pleasant rounding to the recovery of an important member of a family, deceptive it surely is: as, for instance, a father gets well from severe illness because his filial son cuts a piece of flesh from his arm and puts it in the decoction pot to impart special virtue to it. Every year the Peking Gazette gravely makes an honorable mention of filial sons and dutiful daughters-in-law who have thus maimed themselves in their loving devotion to their parents. The efficacy of the human flesh in this connection is widely believed in by all classes here.

In the reign of Hein Fung in the early fifties there was a local rebellion in the Shanghai city. During that atrocious period many a man was slaughtered and butchered and his gall bladder was taken out and invariably swallowed by some savage chieftain with the idea that it would brace up his courage—this latter being said to reside in the gall. The Chinese word for bravery means a large gall.

In the history of the Three Kingdoms mention is made of a distinguished general, who, on an eye being struck out, immediately picked it up and said: "This is made of my father's essence and my mother's blood, I dare not throw it away," and forthwith he swallowed it.

These are instances showing that from time immemorial the people of China have a notion of the peculiar virtue of the human body. And not only so, but even excrementitious and effete matters are sometimes used. Thus, solid fæces are selected and moulded into the shape and size of a chestnut and hermetically sealed in a jar and buried in the ground for a number of years. Then they are taken out again, when they have a gray color and devoid of smell, and they are then carefully covered with gilt and stowed away to be called for by some eccentric medico, when they are euphemistically denominated "Golden Beans." Their ultimate destination is the decoction pot, and the patient knows nothing of it while taking it. A human placenta is sometimes cleaned, cut up in pieces and given to a white duck to feed, and after a few days the latter is killed, its insides taken out and thrown away and the duck is cooked and given to the consumptive to take. Cat's placenta is roasted to ashes on a tile and taken in a warm drink, also for consumption. Young boy's urine is taken in samshu and sugar, though parents generally object to this, having an idea that it will have a reflex action in shortening such a boy's life. Of course there are many other things infinitely more unspeakable that are, or may be used, in the despairing hope of life, but the above I know to be facts from personal observation.

But are there no Chinese drugs and remedies that are worthy to be rescued from oblivion? Certainly there are; indeed many Chinese drugs and remedies have already been naturalized in the Western pharmacopœias, such as rhubarb, camphor, mercury, musk, etc.; and there are some empirical methods that will surely reward a trial, but are either not known or are ignorantly sneered at because they are of Chinese origin, for instance: When a child has eaten too much or otherwise brought on discomfort of the abdomen, nothing is so effective as to put ¼ or 1 oz. of pi-siau in a thin cotton band and apply to the abdomen. By the next morning the stuff has almost entirely disappeared, very likely by absorption, evaporation or mechanical loss; the abdomen has subsided and the child is well. This stuff is native sulphate of soda, sold in all the Chinese drug-shops for this purpose alone. This is a pleasant way of using a cathartic, which it most certainly is. It is easily done, and effective, and done, too, all over China. Another empirical method is that of scraping or pinching the skin for mild cholera, sunstroke, etc. This consists of a cash scraping the back till it is striped like a zebra—only in red. It looks barbarous, but its effect is instantaneous. In the sickly months of the summer, when one feels out of sorts, with perhaps a touch of colic, then let his spine be first scraped clear down to the lumbar region, then down the median line in front and on either side of the ribs he will feel the charm right away. Of course, this is nothing more than counter-irritation, it brings the blood to the surface and is redistributed. But no medicine in the pharmacopœia can give such rapid and happy effects as this under the circumstances.

A milder method is that of pinching the skin of the neck till it is covered with red vertical stripes. There can be no mistake in these methods being useful, as I have experienced them myself many times in these nearly twenty years. With the exception of these few gleams of phosphorescence, it is all darkness in the medical horizon of China.

But thanks to the self-denying efforts of the medical missionaries of all denominations, the healing art is now being taught in many parts of the empire. In the north there are medical students attached to the St. Luke's Hospital in Shanghai, the hospital at Soochow and also in the school of the Presbyterian Mission in Shantung. The latest additional effort is the medical college in Tientsin under the immediate patronage of the Viceroy Li.

Such a medical school will therefore confer an incalculable blessing on China. The Chinese may blindly reject the Christian religion, but they have never refused the offices of the Christian physician; some will receive Christianity; but all will accept medicine.

